# Urban environment, health, and equity: A population-based study of symptoms and annoyances related to air pollution, traffic noise, and greenness

**DOI:** 10.1097/EE9.0000000000000516

**Published:** 2026-08-26

**Authors:** Elodie Eiffener, Andrei Pyko, Östen Axelsson, Rachel Murekatete, Anne-Sophie Merritt, Antonios Georgelis, Olena Gruzieva, Mare Lõhmus, Niklas Andersson, Kristina Eneroth, Mikael Ögren, Marco Pretto, Lorenzo Dorbolò, Petter Ljungman, Anna Bergström, Charlotta Eriksson

**Affiliations:** a Institute of Environmental Medicine, Karolinska Institutet, Stockholm, Sweden; b Centre for Occupational and Environmental Medicine, Region Stockholm, Stockholm, Sweden; c ISGlobal, Barcelona Institute for Global Health, Barcelona, Spain; d Universitat Pompeu Fabra, Barcelona, Spain; e SLB Analys, Environment and Health Department, Stockholm stad, Stockholm, Sweden, currently at COWI AB, Gothenburg, Sweden; f Department of Occupational and Environmental Medicine, Sahlgrenska University Hospital, Gothenburg, Sweden; g Occupational and Environmental Medicine, School of Public Health and Community Medicine, Institute of Medicine, University of Gothenburg, Gothenburg, Sweden; h Dipartimento Politecnico di Ingegneria e Architettura, University of Udine, Udine, Italy; i Department of Cardiology, Danderyd Hospital, Stockholm, Sweden.

**Keywords:** Air quality, Greenspace, Noise, Built environment, Environmental disparities, One Health

## Abstract

**Background:**

Urban residents are exposed to multiple environmental stressors, but robust evidence on inequities in environmental exposure and associated health and well-being outcomes remains limited.

**Aim:**

To assess inequities in residential exposure to air pollution (PM_2.5_, PM_10_, and NO_2_), traffic noise (road, railway, and aircraft) and greenness (NDVI within 300 m), and to characterize associations with self-reported symptoms and annoyances.

**Methods:**

The study population comprised 46,282 adults (18–84 years) residing in Stockholm County, Sweden, who responded to the Swedish National Environmental Health Survey 2023. Residential exposure (dichotomized) was based on geocoded address coordinates and stratified by socioeconomic status (SES) and building type. Logistic regression estimated adjusted odds ratios (OR) with 95% confidence intervals (95% CI) and tested effect modification via interaction terms.

**Results:**

Apartment residents were more often exposed to air pollution and noise and had less surrounding greenness than house residents. SES-stratified analyses revealed exposure-specific patterns, but no consistent overall trends. Air pollution exposure was associated with perceived poor outdoor air quality (NO_2_: OR = 3.22; 95% CI = 2.75–3.79), traffic noise with, for example, high noise annoyance (road traffic: OR = 4.59; 95% CI = 4.02–5.26), and greenness, for example, with lower perceived indoor heat stress during summer (OR = 0.83; 95% CI = 0.79–0.88). Self-reported health and well-being varied by SES and building type.

**Conclusions:**

Our study reveals concentrated environmental stressors in dense residential areas and highlights multidimensional environmental inequities. Environmental exposure was associated with self-perceived health and well-being, underscoring the need for integrated, system-based policies, such as One Health, to promote sustainable urban well-being.

**What this study adds**

Environmental conditions in cities can affect people’s health and well-being in different ways. This study demonstrates that apartment living tends to coincide with locations and urban forms where multiple environmental exposures co-occur. Furthermore, it suggests that environmental inequity is multidimensional and context specific, rather than systematically disadvantaging single socioeconomic groups across all exposures. We found several statistically significant environmental health associations, but the participants’ perceptions varied according to SES and building type. Our findings show that urban environmental health associations are complex and shaped by both the physical environment and social factors, highlighting the need for urban policies to consider integrated approaches.

## Introduction

More than half of the world’s population currently lives in urban areas, with projections showing that this will increase to approximately two-thirds by 2050.^1^ In Sweden, almost 90% of the population already resides in urban areas, with approximately 97% in Stockholm County, the highest proportion among Swedish counties.^2^ Although the county is predominantly urban in terms of population distribution, it also includes substantial nonurban land areas, including forest and archipelago environments.

Urbanization is not a new phenomenon; however, contemporary urban life differs significantly from that of previous decades or centuries.^2^ Rapid population growth, increased urban density, intensified transportation, and widening economic disparities shape modern cities and their living conditions,^2,3^ with these developments posing complex challenges for urban inhabitants and their health.^3^ Air pollution, noise, and limited access to greenspaces are some examples of prevalent urban stressors. Concurrently, climate change-related factors, such as heat, floods, and the (re-)emergence of infectious diseases, as well as social segregation and environmental health inequities, pose additional threats to city dwellers’ health and well-being.^2,3^

The adverse health effects of environmental stressors are widely established, including associations between air pollution and respiratory and cardiovascular health,^4,5^ traffic noise and ischemic heart disease,^6^ heatwaves and all-cause mortality^7^ and the complex relationship between green and blue spaces and mental health.^8^ Despite this, there remains a need for large-scale studies examining the patterns of people’s perceptions of their residential environment and information, which is vital for sustainable urban planning and decision-making. Perception of environmental exposures may provide insight into underlying factors and can influence health outcomes, for example, by shaping psychological stress responses and behavioral adaptations to environmental stressors.^9^

Environmental health inequity refers to unequal exposure to adverse environmental factors (e.g., heat, air pollution, traffic noise, and overcrowding) and the often consequential uneven distribution of related health risks and symptoms across population groups, while accounting for individual circumstances.^10^ Addressing health inequities requires recognizing diversity and acknowledging that long-standing structural social and health inequities place certain groups at greater risk.^11^ Higher exposures to environmental hazards are often attributed to socioeconomic deprivation or geographic location; however, specific determinants of these inequities and their variation across population groups remain insufficiently characterized.^10^ As the European Environment Agency has noted, those most impacted are typically individuals already disadvantaged because of factors such as age, health status, or socioeconomic position, with children, older adults, and people with limited economic resources often disproportionally exposed to urban environmental stressors.^12^ Moreover, factors like building type (e.g., apartment building versus house) may also contribute to environmental health inequities, for instance, through increased exposure to indoor air pollution, noise, or higher temperatures in apartment residents.^13,14^

In global comparison, the quality of life among European citizens is relatively high.^12^ According to Eurostat, Swedish residents report among the lowest levels of pollution, grime, or other environmental problems in the European Union (EU); even in urban areas, fewer than 10% of the population reported being affected by environmental hazards in 2023.^15^ Nevertheless, environmental health inequities across regions and social groups in Europe persist.^12^ In 2023, people with an increased risk of poverty experienced greater disturbance from pollution, grime, and noise compared with those at a lower risk of poverty in the EU.^15^ Moreover, lower-income groups have been shown to have reduced access to greenspace and higher exposure to air pollution, illustrating an “inequality-driven variation in exposure levels.”^3,16^ Hence, on a large scale, these patterns suggest that marginalized communities often experience a disproportionate environmental health burden. At the local level, however, recent studies performed in Sweden indicate a more heterogeneous and context-dependent pattern, with associations varying by environmental exposure, socioeconomic indicator, and population group.^17,18^ For instance, Stucki et al^17^ showed that within urban areas in Uppsala County, adjacent to Stockholm County, Swedish-born women who were either elderly, unmarried, and well-educated appeared disproportionately exposed to a higher environmental burden. Additionally, Azzouz et al^18^ found that neighborhood socioeconomic conditions were strongly associated with environmental burdens in Sweden, highlighting substantial and city-specific environmental inequalities.

In a scoping review on urbanicity and health, Dyer et al^3^ highlighted a persistent “dearth of data” on local-level demographic and socioeconomic characteristics in urban health research. This limits our ability to detect differences in environmental exposures and health across population groups, making it more difficult to identify vulnerable populations and complicating the design of effective preventive interventions. With this study, we contribute to the evidence base on environmental health inequities and related health impacts by using local data from Stockholm County, Sweden. Specifically, we aim to (1) assess whether environmental (multi-) exposures differ across socioeconomic and demographic groups, and (2) characterize the subsequent associations with self-reported symptoms and annoyances.

## Methods

### Study population

The study population is based on the respondents to the Swedish National Environmental Health Survey from 2023. This survey is conducted every 4 years by the Public Health Agency of Sweden and is sent to a random sample of Swedish residents, alternating between surveys targeting children and adults.^19^ Typically, in the adult survey, each of the 21 counties in Sweden is represented with samples of 500 adults aged 18–84 years who have been registered as residents in Sweden for at least 5 years, with additional densification sampling in selected counties and municipalities.^20^ In 2023, extra densification was done in Stockholm County. Environmental health survey results help county administrative boards, regions, and municipalities compare regional conditions with national trends and support environmental protection, public health efforts, and community planning. This is done, for instance, via national or regional environmental health reports and an online statistical visualization tool “Folkhälsostudio.”^19,21^

Of the 235,091 individuals initially invited to participate in 2023, 37.7% responded, corresponding to 88,725 participants, with item-specific nonresponse ranging from 0.3% to 8.7%. Almost half of all invited individuals were residents of Stockholm County because of the extra densification efforts in 2023. The survey collected cross-sectional data on self-reported health, including general health status, chronic diseases, stress, and annoyances, as well as perceptions of environmental exposures and behaviors, such as perceived air quality and noise, frequency of green space visits, and sleep quality. Survey data were subsequently linked to register data, primarily on sociodemographic characteristics and residential address coordinates. Exposures and outcomes were dichotomized using established thresholds from international guidelines, previous studies, or local data to allow clinically and policy-relevant interpretation and comparability across exposures.

Based on these data, we established the Stockholm Environmental Health Cohort, a population-based cohort intended for longitudinal follow-up and comprising survey respondents residing in Stockholm County (N = 46,282). The present study reports cross-sectional findings from the 2023 baseline survey and describes the study sample that forms the basis of the subsequent cohort. Ethical approval was granted by the Swedish Ethical Review Authority, Stockholm Region (Dnr. 2024-08093-01). Participants gave informed consent to participate in the study by responding to the survey.

### Exposures

In this study, we used three main categories of environmental exposures: air pollution (particulate matter with an aerodynamic diameter <2.5 (PM_2.5_), <10 µm (PM_10_), and nitrogen dioxide (NO_2_)), traffic noise (road, railway, aircraft), and greenness (Normalized Difference Vegetation Index (NDVI) within 300 m). We selected air pollution, traffic noise, and residential greenness a priori because they represent major, policy-relevant dimensions of the urban residential environment in Stockholm County. Together, these domains capture both environmental stressors and health-promoting resources and allow assessment of whether urban environmental burdens and amenities are distributed differently across population groups. The selection was based on established environmental health evidence, regional monitoring priorities, and previous environmental health reporting in Stockholm County.^4–8,21^ We used a directed acyclic graph to specify assumed relationships between the exposures, outcomes, and potential confounders (Figure S0, https://links.lww.com/EE/A454). All exposures were assessed objectively at participants’ residential addresses, using geocoded address coordinates. Exposure assessment was done according to state-of-the-art and validated methods. A detailed description of the exposure assessment can be found in Table S0, https://links.lww.com/EE/A454. To facilitate the analysis of associations with self-reported symptoms and annoyance outcomes, all environmental exposures were dichotomized into high and low exposure categories based on established thresholds from international guidelines, previous literature, and local reports. For air pollution, exposure cutoffs were defined at the pollutant-specific median values, with values higher than or equal to the median classified as high exposure; given considerably low exposure levels, predominantly below World Health Organization (WHO) air-quality guideline cutoffs. For traffic noise, we used the WHO cutoffs, which consider high exposure as ≥53 dB L_den_ for road traffic, ≥54 dB L_den_ for railway traffic, and ≥45 L_den_ for aircraft noise. High greenness exposure was defined as NDVI ≥0.45, based on previous literature and accumulated regional experience, for example, from local environmental health reporting.^21,22^

### Outcomes

The selection of outcome variables was intended to provide a broad assessment of potential health and well-being impacts of multiple environmental exposures and was guided by a priori hypotheses based on previous epidemiological evidence and the availability of relevant data in the National Environmental Health Survey.^20^ All outcomes were self-reported and derived from the survey questionnaire. Several outcomes were constructed by combining responses from multiple survey questions; detailed variable definitions and coding are provided in Table S1, https://links.lww.com/EE/A454. For air pollution exposures, we investigated associations with good general health (yes/no), perceived poor outdoor air quality (yes/no), symptoms from eyes, nose, and/or throat (yes/no), bronchitis (yes/no), and asthma (yes/no). For traffic noise exposures, outcomes included good general health (yes/no), high noise annoyance (yes/no), disturbance of rest and relaxation (yes/no), general stress symptoms (e.g., headache and/or fatigue) (yes/no), sleep disturbance because of traffic (not source-specific; yes/no), perceived high indoor temperatures during summer (yes/no), nuisance while on terrace or balcony (yes/no), as well as much time spent outdoors on terrace or balcony (yes/no). High noise annoyance was assessed separately for road, railway, and aircraft noise. For greenness exposure, we examined associations with good general health (yes/no), general stress symptoms (e.g., headache and/or fatigue) (yes/no), symptoms from eyes, nose, and/or throat (yes/no), pollen allergy (yes/no), frequent greenspace visits (yes/no), perceived high indoor temperatures during summer (yes/no), and amount of time spent outdoors on the balcony or terrace in summer (yes/no).

### Covariates

Our adjustment strategy was based on a common causal framework (illustrated in Figure S0, https://links.lww.com/EE/A454). Consequently, we used the same set of covariates across all exposure-outcome models. Covariates were selected a priori as potential confounders based on existing literature and causal assumptions represented in the directed acyclic graph, while also considering data availability.^20,21,23^ Individual and lifestyle characteristics included age (18–39, 40–59, 60–84 years), sex (men/women), and smoking status (current/former/never). Socioeconomic status (SES) was represented by the highest attained education level (elementary school/upper secondary school/university), country of birth (domestic/foreign), civil status (married/not married), and disposable household income (continuous, SEK). As area-based covariates, municipality of residence and area-level income at the DeSO (“Demografiska statistikområden”) level were included in the main models.^24^ DeSOs are small-area statistical units in Sweden comprising approximately 700–3,000 inhabitants that provide insights into statistics at a low regional level, with information originating from both registers and the survey. For stratified and dichotomized analyses, university degree was classified as high education, and household income above the median (>652,552 SEK) was classified as high income.

### Statistical analysis

We used descriptive analyses to characterize the study population, including only individuals with non-missing information on the relevant exposure and/or outcome variables. Continuous variables were presented as medians with interquartile ranges (IQRs) and categorical variables as frequencies and proportions (n/N). Based on their DeSO codes, we allocated the study participants as living in urban, suburban, or rural areas of Stockholm County. Because air pollution, traffic noise, and greenness are partly shaped by the same underlying urban form and transport infrastructure, these exposures were expected to be correlated. We therefore examined pairwise Pearson correlations between exposure variables to describe the degree of exposure cooccurrence in the study sample. To broadly capture environmental exposure differences across major population groups, we summarized the proportion of participants exposed to high levels of air pollution, traffic noise, and greenness overall and stratified by income (high/low), education (high/low), country of birth (domestic/foreign), and building type (house/apartment building). Pearson’s chi-squared tests were used to compare proportions across groups.

To assess potential selection bias, all analyses were repeated using survey weights provided by Statistics Sweden, designed to account for differential non-response across population subgroups.^20,25^ Thereafter, via multivariate logistic regression models, adjusted for the predefined covariates, we estimated the associations of selected health and well-being outcomes in relation to the respective environmental exposures.

In sensitivity analyses, we excluded area-based income at the DeSO level as a covariate from the main model. Additionally, in selected key exposure-outcome associations, we included municipality as a random effect. Finally, we reanalyzed selected associations with environmental exposures modeled as continuous variables using natural cubic splines (3 degrees of freedom). Likelihood ratio tests comparing the spline and linear models were used to assess potential nonlinearity. The significance level for all analyses was set at alpha = 0.05. Effect modification was explored for targeted exposure-outcome associations by income, education, country of birth, and building type using interaction terms. The interaction analyses were limited to one outcome per exposure, selected based on both the relevance for regional policymaking, as well as the strength and significance of associations in the logistic regression analyses. We used CRAN R (version 4.3.2) for all the statistical analyses.

## Results

Study population characteristics are presented in Table 1 and indicate reasonably even sex and age distributions, with a slightly higher proportion of participants aged 60 years or older (46%). Around half of our study population lived in an apartment building. Similarly, about half held a university degree, half had never smoked, and half were married. Although many participants lived in apartments, only 19% were rental tenants. Most respondents were born in Sweden (83%) and had a good economic status. The majority of our sample population lives in urban areas (90.1%), while 3.3% of the participants lived in suburban areas and 6.6% in rural areas.

**Table 1.** Study population characteristics

Characteristics	N^a^	Overall^b^, N = 46,282
Sex	46,282	
Men		20,753 (45%)
Women		25,529 (55%)
Age in years	46,282	
18–39		9,455 (20%)
40–59		15,760 (34%)
60–84		21,067 (46%)
Country of birth	45,926	
Domestic		37,975 (83%)
Foreign		7,951 (17%)
Highest education level	46,094	
Elementary school		4,642 (10%)
Upper secondary school		19,277 (42%)
University		22,175 (48%)
Smoking	46,157	
Current		3,815 (8.3%)
Former		16,280 (35%)
Never		26,062 (56%)
Civil status	46,282	
Married		22,744 (49%)
Not married		23,538 (51%)
Disposable household income (SEK)	46,282	
0–299,999		8,841 (19%)
300,000–599,999		15,433 (33%)
600,000+		22,008 (48%)
Building type	44,665	
Apartment building		24,043 (54%)
House		20,622 (46%)
Housing form	44,665	
Rental		8,676 (19%)
Right of residence		16,840 (38%)
Ownership		19,149 (43%)

a Less than 9% missing for each variable.

b n (%).

Median residential air pollution exposure levels were 4.47 μg/m^3^ for PM_2.5_, 8.68 μg/m^3^ for PM_10_, and 7.23 μg/m^3^ for NO_2_, which roughly correspond to measured levels in urban background air in central Stockholm (Figure S1, https://links.lww.com/EE/A454). Median noise exposure levels were 56.4 dB L_den_ for road traffic, 54.8 dB L_den_ for railway traffic, and 30.7 dB L_den_ for aircraft noise. The median exposure level of NDVI was 0.57. Figure S2 (https://links.lww.com/EE/A454) illustrates the correlation between the different exposure variables. According to Cohen (1998), the correlation between the different exposures is moderate to high, with inverse correlations for greenness and no correlation of railway noise with any other exposure variable.^26^

Table 2 presents the proportion of people exposed above the predefined cutoffs for each environmental factor, stratified by income, education, country of birth, and building type. Participants with a university education were more likely to have air pollution exposure levels above the median for PM_2.5_, PM_10_ and NO_2_ (57%) than participants without a university degree (43–44%). Exposure proportions were also substantially higher among residents of apartment buildings (59–71%) than among those living in houses (including detached, semidetached, and row houses) (25–41%). Patterns were less consistent for income and country of birth, although slightly higher proportions of exposure to PM_10_ and NO_2_ were observed among individuals with a foreign background than among those born in Sweden (53% vs. 49%).

**Table 2.** Proportion of people exposed above respective cutoff values, stratified by income, education, country of birth, and building type

Exposure		Income^a^		Education^a^		Country of birth^a^		Building type^a^	
	Overall	High^e^ N = 23,137	Low^e^ N = 23,137	High^f^ N = 22,175	Low^f^ N = 23,919	Domestic N = 37,975	Foreign N = 7,951	House N = 20,662	Apartment building N = 24,043
Air pollution^b^									
PM_2.5_	23,260 (50%)	12,265 (53%)	10,991 (48%)	12,622 (57%)	10,546 (44%)	19,102 (50%)	3,949 (50%)	8,470 (41%)	14,064 (59%)
PM_10_	23,144 (50%)	11,517 (50%)	11,621 (50%)	12,570 (57%)	10,461 (44%)	18,709 (49%)	4,225 (53%)	6,173 (30%)	16,119 (67%)
NO_2_	23,134 (50%)	11,274 (49%)	11,854 (51%)	12,635 (57%)	10,384 (43%)	18,683 (49%)	4,242 (53%)	5,114 (25%)	17,115 (71%)
Traffic noise^c^									
Road	29,828 (64%)	13,839 (60%)	15,984 (69%)	14,830 (67%)	14,853 (62%)	23,893 (63%)	5,667 (71%)	8,188 (40%)	20,527 (85%)
Railway	3,413 (7.4%)	1,596 (6.9%)	1,816 (7.8%)	1,746 (7.9%)	1,649 (6.9%)	2,700 (7.1%)	680 (8.6%)	709 (3.4%)	2,550 (11%)
Aircraft	1,629 (3.5%)	754 (3.3%)	875 (3.8%)	742 (3.3%)	881 (3.7%)	1,296 (3.4%)	315 (4.0%)	628 (3.0%)	953 (4.0%)
Greenness^d^									
NDVI	37,107 (80%)	18,788 (81%)	18,311 (79%)	16,998 (77%)	19,956 (83%)	30,387 (80%)	6,451 (81%)	20,397 (99%)	15,662 (65%)

a n (%), Pearson’s Chi-squared test, less than 9% missing for each variable.

b Air pollutants as binary variables. Cutoffs (high) in μg/m^3^ at respective medians—PM_2.5_ ≥ 4.47, PM_10_ ≥ 8.68, NO_2_ ≥ 7.23.

c Noise as binary variables. Cutoffs (high) in dB L_den_, according to WHO limits for traffic noise—road ≥53, railway ≥54, aircraft ≥45.

d Greenness as a binary variable. Cutoff (high) in Normalized Difference Vegetation Index (NDVI) at ≥0.45.

e Low income = below or equal to median (≤652,552); high income = above median (>652,552).

f Low education = no university degree; high education = university degree.

Overall, 64% of participants were exposed to road traffic noise levels above the WHO recommended guideline value of 53 dB L_den_. Higher exposure proportions were observed among individuals with low income, foreign background, apartment residency, and higher education (Table 2). The largest contrast was observed by building type, with 85% of apartment residents exposed above the guideline compared with 40% of those living in houses. Similar patterns were observed for railway noise, although overall exposure prevalence was considerably lower (7.4%). Aircraft noise exposure was rare, with only 1,629 (3.5%) participants being exposed, with no conclusive patterns across the strata.

Concerning greenness, 80% of the study population were exposed to an NDVI value ≥0.45 (Table 2). The strongest differences were observed for building type, with 99% of the people in houses being exposed to high greenness levels compared to 65% of people in apartment buildings. Similarly, noise, education, and income presented opposing patterns regarding greenness, with slightly lower proportions of high greenness exposure among people with low income but high education.

The results from the weighted study sample, which accounted for potential selection bias (Table S2, https://links.lww.com/EE/A454), confirmed the general trends and differences between population groups and indicated an even higher overall proportion of exposure to environmental hazards.

The distribution of health and well-being outcomes in our sample is presented in Table S3 (https://links.lww.com/EE/A454). Results from the logistic regression analyses, adjusted for socioeconomic factors, are shown in Table 3. Concerning air pollution, statistically significant associations were found between exposure above the median to all air pollutants, respectively, and perceived poor outdoor air quality, with the strongest association for NO_2_ (OR = 3.20; 95% CI = 2.73–3.76). Positive, albeit nonstatistically significant associations were also observed between exposure to air pollution and bronchitis. However, no consistent associations were observed for good general health, symptoms from the eyes, nose, and/or throat, or asthma.

**Table 3.** Associations (OR and 95% CI) between environmental exposures and indicators of health and well-being (all)

	Air pollution^a^^,^^b^(μg/m^3^)			Traffic noise^a^^,^^c^(dB L_den_)			Greenness^a^^,^^d^(NDVI)
Outcomes	PM_2.5_	PM_10_	NO_2_	Road	Railway	Aircraft	NDVI
Good general health	1.00 (0.94–1.06)	1.06 (0.99–1.14)	1.03 (0.96–1.11)	0.96 (0.91–1.01)	0.98 (0.90–1.07)	1.11 (0.98–1.26)	1.00 (0.94–1.07)
Poor outdoor air quality	1.45 (1.29–1.63)	2.70 (2.33–3.15)	3.20 (2.73–3.76)	–	–	–	–
Symptoms from eyes, nose, and/or throat	0.97 (0.92–1.03)	0.99 (0.93–1.06)	1.05 (0.99–1.12)	–	–	–	0.90 (0.85–0.96)
Bronchitis	1.05 (0.86–1.29)	1.06 (0.86–1.32)	1.13 (0.91–1.40)	–	–	–	–
Asthma	1.04 (0.95–1.15)	0.98 (0.89, 1.10)	1.00 (0.90–1.12)	–	–	–	–
High noise annoyance	–	–	–	4.59 (4.02–5.26)	8.37 (6.93–10.1)	7.28 (6.27–8.45)	–
Disturbance of rest and relaxation	–	–	–	1.77 (1.60–1.96)	1.37 (1.22–1.55)	1.81 (1.54–2.11)	–
Sleep disturbance by traffic	–	–	–	1.78 (1.62–1.95)	1.47 (1.32–1.65)	1.43 (1.22–1.67)	–
Nuisance while on terrasse or balcony	–	–	–	2.32 (2.06–2.62)	1.75 (1.55–1.98)	2.27 (1.93–2.67)	–
General stress symptoms (e.g. fatigue, headache)	–	–	–	1.01 (0.96–1.06)	1.05 (0.97–1.13)	0.94 (0.84–1.05)	0.96 (0.91–1.01)
Much time spent outdoors on balcony or terrasse (in summer)	–	–	–	0.34 (0.31–0.38)	0.74 (0.67–0.82)	1.24 (1.05–1.45)	3.24 (3.01–3.48)
Pollen allergy	–	–	–	–	–	–	1.01 (0.96–1.07)
Frequent greenspace visits	–	–	–	–	–	–	1.14 (1.06–1.22)
High indoor temperatures (in summer)	–	–	–	1.10 (1.05–1.16)	1.17 (1.08–1.27)	0.97 (0.86–1.09)	0.81 (0.76–0.85)

a Odds ratios with confidence intervals (95%).

Logistic regression models adjusted for sex, age, country of birth, household income, education level, civil status, smoking, municipality, and area-based income (DeSO). Less than 9% missing for each variable.

b Air pollutants as binary variables. Cutoffs (high) in μg/m^3^ at respective medians—PM_2.5_ ≥ 4.47, PM_10_ ≥ 8.68, NO_2_ ≥ 7.23.

c Noise as binary variables. Cutoffs (high) in dB L_den_, according to WHO limits for traffic noise—road ≥53, railway ≥54, aircraft ≥45.

d Greenness as a binary variable. Cutoff (high) in Normalized Difference Vegetation Index (NDVI) at ≥0.45.

Traffic noise exposure above the WHO guideline levels for road traffic, railway, and aircraft noise was associated with high noise annoyance, disturbance of rest and relaxation, sleep disturbances, and nuisance when spending time on the terrace or balcony (Table 3). Furthermore, we observed inverse associations between both road traffic and railway noise (road: ORs = 0.34; 95% CI = 0.31–0.38; railway: 0.74; 95% CI = 0.67–0.82) and time spent outdoors on the terrace or balcony during summer. Aircraft noise exposure was, however, positively associated with time spent outdoors. High traffic noise from roads and railways, but not aircraft, was positively associated with perceived high indoor temperatures during summer (ORs = 1.10; 95% CI = 1.05–1.16; ORs = 1.17; 95% CI = 1.08–1.27; and ORs = 0.97; 95% CI = 0.86–1.09, respectively). However, no associations were observed between traffic noise and general stress symptoms, such as fatigue or headache.

High levels of residential greenness were associated with more time spent outdoors on the balcony or terrace during summer, frequent greenspace visits, and a lower likelihood of reporting high indoor temperatures during summer. Participants with high residential greenness were 19% less likely to report high indoor temperatures during summer compared with those with low levels of greenness. We also observed an inverse association between greenness and symptoms from the eyes, nose, and throat (OR = 0.90; 95% CI = 0.85–0.96), while no association was found with pollen allergy. Furthermore, in this study, greenness was not associated with general health, nor with stress symptoms.

Sensitivity analyses excluding area-level (DeSO) income as a covariate yielded results broadly consistent with the main findings (Table S4, https://links.lww.com/EE/A454). Similar findings were also obtained when including the municipality as a random effect in the main model, instead of as a covariate. Sensitivity analyses modeling one representative association for each environmental exposure as a continuous variable demonstrated evidence of nonlinearity for PM_2.5_ (likelihood ratio test, *P* < 0.001), road traffic noise (*P* = 0.007), and greenness (*P* < 0.001).

**Figure 1.**
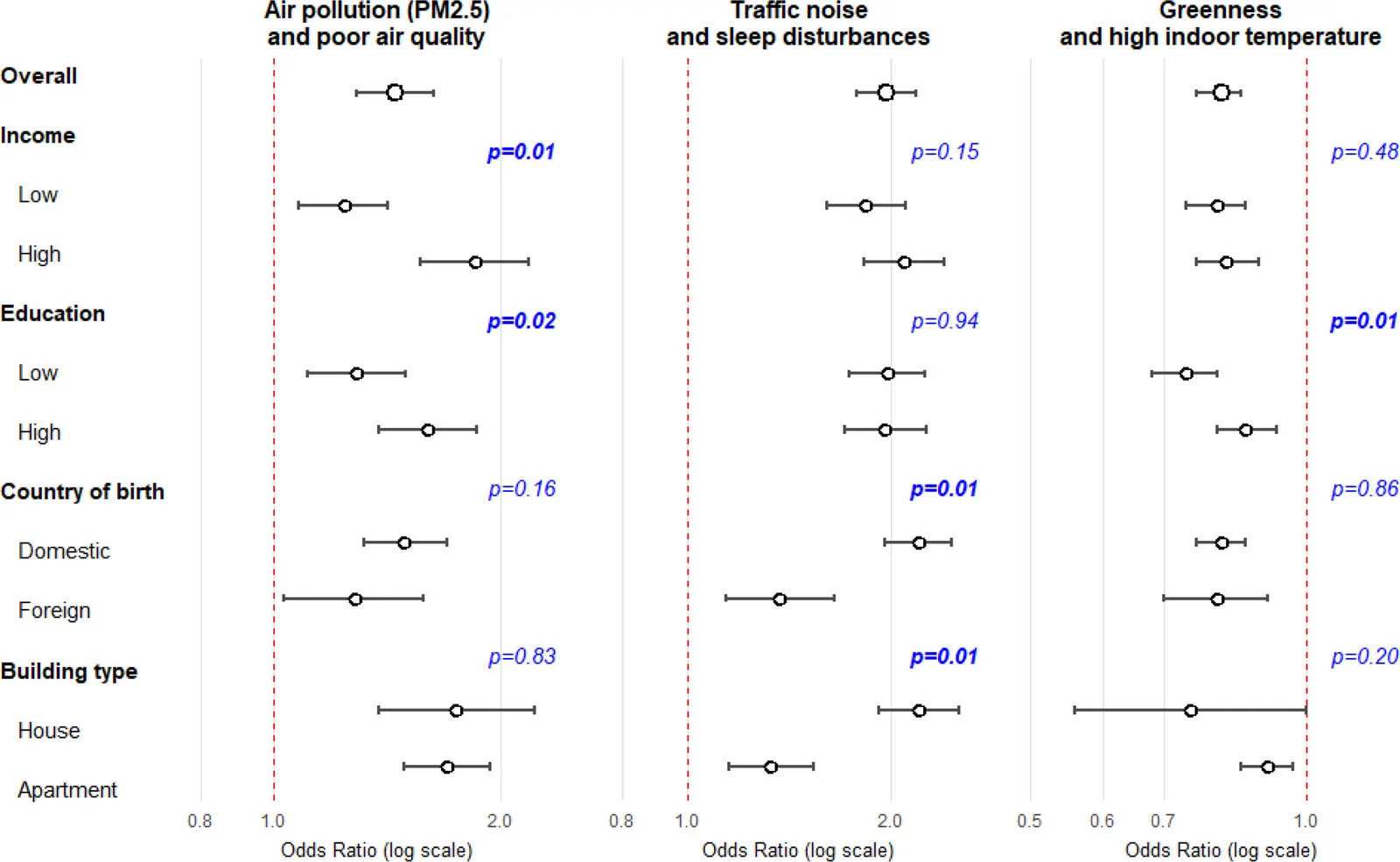
Effect modification analyses in relation to income, education, country of birth, and building type. (a) Air pollution (PM2.5) and perceived poor outdoor air quality. (b) Road traffic noise and sleep disturbance by traffic. (c) Greenness (NDVI) and high indoor temperatures in summer.

Figure 1 presents results from the explorative analyses of effect modification by income, education, country of birth, and building type for three selected exposure-outcome associations. The association between PM_2.5_ and perceived poor outdoor air quality (Figure 1A) differed by income and education, with stronger associations observed among participants with higher income and university education. For combined traffic noise and sleep disturbance (Figure 1B), we observed stronger associations among the participants born in Sweden and those living in a house compared with individuals with a foreign background and apartment residents. For greenness and perceived high indoor temperature (Figure 1C), associations differed by education level, with higher-educated participants reporting higher perceived indoor temperatures in relation to greenness compared with those with a low educational background.

## Discussion

### Summary of findings

The results of this study indicate that the ambient air quality in Stockholm County is generally good, with annual median exposure levels among our study participants below both the WHO Global Air Quality Guidelines and the Swedish environmental targets.^27,28^ In contrast, exposure to road traffic noise above the WHO guideline levels was common (64%).^29^ Despite the fact that the majority of the sample population lived in urban areas (90.1%), most of them (80%) resided in areas with high residential greenness (NDVI ≥ 0.45). Across all exposure domains, residents of apartment buildings were more frequently exposed to air pollution and road and railway noise and had lower surrounding greenness compared with residents of houses; however, the distribution of environmental exposures did not follow a consistent socioeconomic gradient. Instead, patterns varied by exposure type and socioeconomic indicator, with different groups being more exposed depending on the specific environmental factor considered. Environmental exposures were associated with several symptoms and annoyances; for example, traffic noise was linked to high annoyance, sleep and relaxation problems, reduced time spent outdoors, and perceived indoor heat problems during summer. Conversely, greenness was associated with increased time spent outdoors and reduced indoor heat problems during summer. Self-reported environmental responses varied by SES and building type.

### Findings in context

Urban design shapes both the distribution of environmental exposures and access to health-promoting resources, such as green and blue spaces or walkable infrastructure, which are often unequally distributed across population groups.^12,30,31^ Unequal exposure to those determinants constitutes a key pathway through which urban environments contribute to environmental health inequity, motivating our assessment of both differential exposures and their associations with health-related symptoms and perceptions. In a theoretical framework developed by Gelormino et al^32^, the urban built environment is proposed to influence human health through three main trajectories: the natural environment (e.g., air quality and climate), the social context (e.g., public spaces and social interaction), and behavioral factors (e.g., physical activity and lifestyle). The authors identify density, functional mix, and availability of public spaces and services as key features of the built environment that shape these pathways and, consequently, health outcomes. Our findings align with the framework in which the urban environment influences health through environmental pathways that are unequally distributed across population groups but without clear sociodemographic trends.

In an international comparison, ambient air pollution levels in Sweden are relatively low, even in urban areas.^15^ The air quality in Sweden has improved over recent decades, and air pollutant levels have remained below EU guidelines for several years.^33^ However, there may be exceedances in certain areas, particularly during springtime when road wear dust levels peak. Population exposure to traffic noise, on the other hand, is common and remains a public health concern.^34^ Increased urbanization, densification, and transport intensity, coupled with relaxed regulatory constraints, are the main drivers of traffic noise exposure in Stockholm County.^21^ Notably, the current Swedish guideline values for road and railway noise in newly built areas are 10–15 dB above the WHO recommendations.^35^ Although approximately 70% of Sweden’s land area is covered by forests,^36^ urban greenness, which is currently high in Stockholm County, faces increasing pressure from ongoing urban development.^21^ At the same time, urban greenspaces are especially important from a changing climate perspective, counteracting many negative risks that come with both urbanization and climate change. The correlation between the environmental exposures in our study was found to be moderate to very high (Figure S2, https://links.lww.com/EE/A454), except for railway noise. This is to be expected in urban areas and in line with previous findings.^37–39^

The multiple exposure burden observed among apartment residents in this study likely reflects differences in urban form and degrees of urbanicity. Compact cities with high population density are predominantly characterized by apartment buildings, often located near transport and services infrastructure. While compact cities are often considered health-promotive and efficient in reducing some environmental exposures,^3^ they may also be a source of pollution and disease. For instance, compactness and high density can, on the one hand, be beneficial for mobility and active transport, thus reducing energy needs and climate impact, but on the other hand, they are often linked with increased pollution concentrations, higher noise levels, less greenspace, and higher temperatures. Based on our descriptive results, there is a clustering of environmental burdens among apartment residents in Stockholm, potentially contributing to inequities in environmental conditions relevant to health and well-being. Although these findings should be interpreted with caution, they underscore the importance of careful consideration of the location and design of new residential buildings in urban planning. They also highlight the need for measures to reduce environmental exposures in these residential environments to prevent adverse effects on human health and well-being. New urban models, such as Superblocks or Low-Traffic-Neighborhoods, that have increasingly gained popularity in European cities after the COVID-19 pandemic, could be promising solutions in this regard.^40^

Despite a relatively good quality of life in Europe, socioeconomic disparities in environmental exposure persist.^10^ Vulnerable groups, including children, the elderly, and people with low income, often experience a disproportionate environmental burden, potentially leading to a double burden of social and environmental disadvantage. While most literature from North America, Africa, Asia, and New Zealand frequently reports higher air pollution exposure among socially disadvantaged groups, research from Europe shows mixed results.^41^ Our results confirm previous findings from Swedish studies that also described heterogeneous patterns of environmental health inequalities, with associations varying by exposure type, SES indicator, and spatial context.^17,18^ As emphasized by Dyer et al,^3^ certain individuals may remain particularly vulnerable to specific environmental exposures even at relatively low exposure levels. This highlights the importance of considering population vulnerability and environmental equity alongside average exposure levels. Ensuring access to healthy environments for all should therefore remain a central objective of urban planning and policymaking.

Air pollution in this study was only associated with self-perceived poor air quality, with NO_2_ showing the strongest associations. NO_2_ is primarily emitted from combustion processes, such as from road traffic, and thus has a major impact on air quality in urban areas. The lack of association between air pollution and respiratory symptoms or diseases, however, contrasts with current scientific evidence, including recent work linking NO_2_ to the global burden of asthma.^27,42^ In addition, a systematic review including studies published between January 1980 and July 2019 showed moderate-to-high confidence in associations between air pollution and asthma onset in both children and adults, acute lower respiratory infections in children, and lung cancer mortality in adults, as well as moderate evidence for respiratory mortality in adults.^43^ Several factors may explain the discrepancy between these findings and our results, including the relatively low air pollution concentrations in Stockholm County, limited exposure contrast within the study population, outcome misclassification because of self-report, a relatively healthy study population, and residual confounding. Although health effects of air pollution have been observed even at relatively low concentrations, such factors may have reduced our ability to detect associations in this setting. Previous findings from Stockholm County have indicated associations between air pollution and respiratory symptoms in adults; however, dating nearly 20 years back, air pollution concentrations have improved considerably since then, likely explaining the discrepancy in findings.^44^

For traffic noise, associations with self-reported annoyance and sleep disturbance are well established,^45,46^ and were broadly confirmed in this study. In contrast, evidence regarding relationships between traffic noise, outdoor activity patterns, and perceived indoor heat is limited. A few previous studies have found that traffic noise may deter walking and reduce physical activity.^47,48^ Our findings support these associations, indicating that noise causes nuisance while on the terrace or balcony and reduces the time spent outdoors. Moreover, in a study from Greater London, nearly one-third of the public greenspaces have been shown to exceed WHO guidelines for noise, with higher levels observed in more central urban areas.^49^ Such conditions may reduce physical activity levels and attenuate the restorative benefits of greenspace use. To our knowledge, no previous study has specifically examined the relationship between traffic noise and perceived indoor heat. Experimental evidence, however, suggests that thermal and acoustic environments interact to influence comfort perceptions. For example, a controlled study from China identified combined thermal and acoustic thresholds beyond which comfort is adversely affected.^50^ Our findings suggest that traffic noise is associated with increased self-perceived indoor heat during summer. This may be part of a single behavioral response to noise, including refraining from spending time outdoors and keeping windows closed. Thus, the observed associations may not represent entirely independent effects. Together, heat and noise exposure may exacerbate sleep disturbances and interact in the etiology of cardiovascular disease; however, more research in this area is needed.

Conversely, our findings further suggest a potential buffering effect of greenness on perceived indoor heat, in line with contemporary evidence. A recent systematic review on community-based heat adaptation interventions postulated that green roofs and green walls can reduce indoor temperatures by up to 2.4°C.^51^ Although such interventions are primarily designed to mitigate outdoor heat, these results indicate substantial potential for indoor cobenefits of nature-based solutions in urban environments. Our study also underscores the importance of residential greenness to stimulate time spent outdoors and frequent greenspace visits. Furthermore, greenness was associated with a reduced risk of reporting symptoms from the eyes, nose, and/or throat, which may partly reflect the influence of greener environments on local air quality. Vegetation can remove airborne pollutants through deposition and uptake processes; however, the magnitude of these effects on local pollutant concentrations is highly context-dependent and varies according to vegetation structure, urban morphology, and meteorological conditions.^52,53^ Recent evidence suggests that while urban greenness can contribute to improved air quality under certain circumstances, its effects on pollutant concentrations and human exposure are often modest compared with reductions achieved through emission control measures.^54,55^ Therefore, other mechanisms, including microclimatic regulation and behavioral pathways, may also contribute to the observed associations.

Lastly, our interaction analyses indicated that SES and building type modified associations between environmental exposures and subjective symptoms and annoyances. This is important to consider in future environmental health surveys. While SES is a well-established determinant of self-reported health, its modifying role in environmental health research remains understudied. Few to no studies have explicitly examined SES and residential characteristics as effect modifiers rather than confounders. However, evidence from the United Kingdom suggests that individuals in more deprived areas may underreport health problems, potentially leading to an underestimation of health inequalities.^56^ Building type has also rarely been studied as an effect modifier in previous epidemiological studies. The underlying mechanisms are difficult to disentangle; however, suggested pathways, examined for instance in the context of indoor air pollution and indoor heat, include the greater likelihood of apartment buildings being located in denser urban areas, often near major roads and associated with a higher overall environmental burden, as well as factors like building physics and layout.^13,14^ These findings highlight the need for careful interpretation of health inequality statistics and resulting public health interventions.

The complex interplay between SES, urban form, individual vulnerability, and environmental exposures in European settings makes the design of public health interventions focused on environmental health equity a difficult task. At the same time, it underscores the need for local, context-specific, inter-, and transdisciplinary policies and actions. Systems-based solutions and integrated approaches, particularly the One Health approach, are indispensable in this context. These are interventions that recognize the interconnections and interdependency between human, animal, and ecosystem health, and that are developed collaboratively across sectors and with stakeholders.^57^ In that sense, environmental health equity cannot be achieved at the cost of animal or ecosystem health. A One Health-inspired transport strategy could, for instance, involve urban planners, transport experts, environmental scientists, public health professionals, and citizen groups to enforce low-emission zones, cycling infrastructure, public transport improvements, and so on, with the aim of reducing air pollution and noise, increasing physical activity, creating green and blue recreation space, lowering greenhouse gas emissions, and improving health and well-being for all. One Health-guided urban decision-making, also summarized under the term “Urban One Health,” is therefore key in maximizing cobenefits and reducing unintended harm of urban (public health) interventions.

### Limitations and strengths

The results of this study need to be interpreted in view of its limitations. First, due to its cross-sectional design, the ability to determine causal inferences is limited. Second, environmental exposures were assessed at residential addresses and did not account for the duration of residence, daily mobility, or time-activity patterns. Moreover, the relatively high correlation between our exposures may raise concerns about whether the estimated coefficients can truly be interpreted as independent effects rather than markers of the broader urban environmental exposure mixture. However, we believe that we mainly modeled distinct exposure-specific health outcomes and pathways (e.g., traffic noise and noise annoyance), although we acknowledge that for some outcomes, for example, general health, the exposure correlation may lead to difficulties in disentangling the exact risk factor. Third, indoor environmental exposures were not investigated, despite adults spending a substantial proportion of time indoors. This may have led to nondifferential misclassification of exposure, potentially leading to a dilution of the associations. Fourth, health and well-being outcomes were self-reported, which may have introduced nondifferential misclassification and attenuated associations. In addition, although we adjusted for central sociodemographic and lifestyle markers, we cannot rule out residual confounding. Fifth, the response rate to the questionnaire was relatively low (37.7%), which raises concerns about selection bias. This was addressed by performing additional analyses in a weighted study sample, which confirmed our main findings. Nevertheless, the socioeconomic status of the sample population appeared higher than in the general population, which may have led to an underestimation of our study results (e.g., median household income in the study sample of 652,552 compared with 423,000 SEK in the general population in 2022).^58^ Moreover, our sample included only residents who had lived in Sweden for at least 5 years at the time of the survey, reflecting a limitation of the data and likely underrepresenting recent migrants. However, our findings may still be generalizable, as recent migrants are a heterogeneous group, including, for instance, individuals from EU and non-EU countries and diverse socioeconomic backgrounds. Finally, the dichotomization of exposures and outcomes may have resulted in some loss of information and reduced granularity compared with continuous or ordinal approaches, which could have attenuated estimated associations.

Simultaneously, this study also inherits various strengths. First, it meets the need for the identification of local-level disparities in environmental exposure and its associated symptoms and annoyances by using high-resolution exposure data coupled with individual-level survey and register data.^3^ Second, the study is based on the hitherto largest population-based environmental health survey ever performed in Sweden, rendering high statistical power and facilitating generalisability of the results. We believe that our environmental health associations are generalizable to other cities and regions with similar urban characteristics and exposure profiles; however, the exposure inequalities are likely to be context dependent and site specific. Third, we were able to control for potential confounding from sociodemographic factors using both survey and register data. Finally, this study adds to the body of evidence of people’s perceptions of their health and well-being in relation to the ambient environment with the aim of capturing the unseized potential of sustainable cities in terms of public health and welfare.

## Conclusion

This study demonstrates that apartment living tends to coincide with locations and urban forms that concentrate multiple environmental exposures. Furthermore, it suggests that environmental inequity is multidimensional and context specific, rather than systematically disadvantaging a single socioeconomic group across all exposures. We observed several adverse health and well-being impacts of environmental exposure, particularly in relation to traffic noise, which was associated with annoyance, sleep and relaxation problems, reduced time spent outdoors, and increased indoor heat problems. Conversely, greenness stimulated outdoor activities and reduced perceived indoor heat. However, self-perceived health differed by SES and building type. The complex interplay among the built environment, social determinants, and health and well-being underscores the need for integrated, transdisciplinary, and system-based policies to support sustainable urban living, as exemplified by the One Health approach.

## Conflict of interest statement

The authors declare that they have no financial conflict of interest with regard to the content of this report.

## Acknowledgments

The Centre for Occupational and Environmental Medicine, Region Stockholm, was responsible for database development and maintenance. The Swedish Public Health Agency was responsible for the distribution and data collection of the National Environmental Health Survey 2023. The authors wish to extend their gratitude to the participants of the National Environmental Health Survey of 2023 which made the study possible, and to the Swedish Public Health Agency for sharing their data.

## Supplementary Material
