# Supplementary material for: Urban environment, health, and equity: A population-based study of symptoms and annoyances related to air pollution, traffic noise, and greenness
Source: Environ Epidemiol. 2026 Aug 26;10(5):e516. doi: 10.1097/EE9.0000000000000516 (PMC13521496; doi:10.1097/EE9.0000000000000516)
Supplement: Supplementary file 1 [file ee9-10-e516-s001.pdf]

SUPPLEMENT

Figure S0. Directed acyclic graph (DAG) illustrating the assumed relationships among exposures, outcomes, confounders, and mediators.

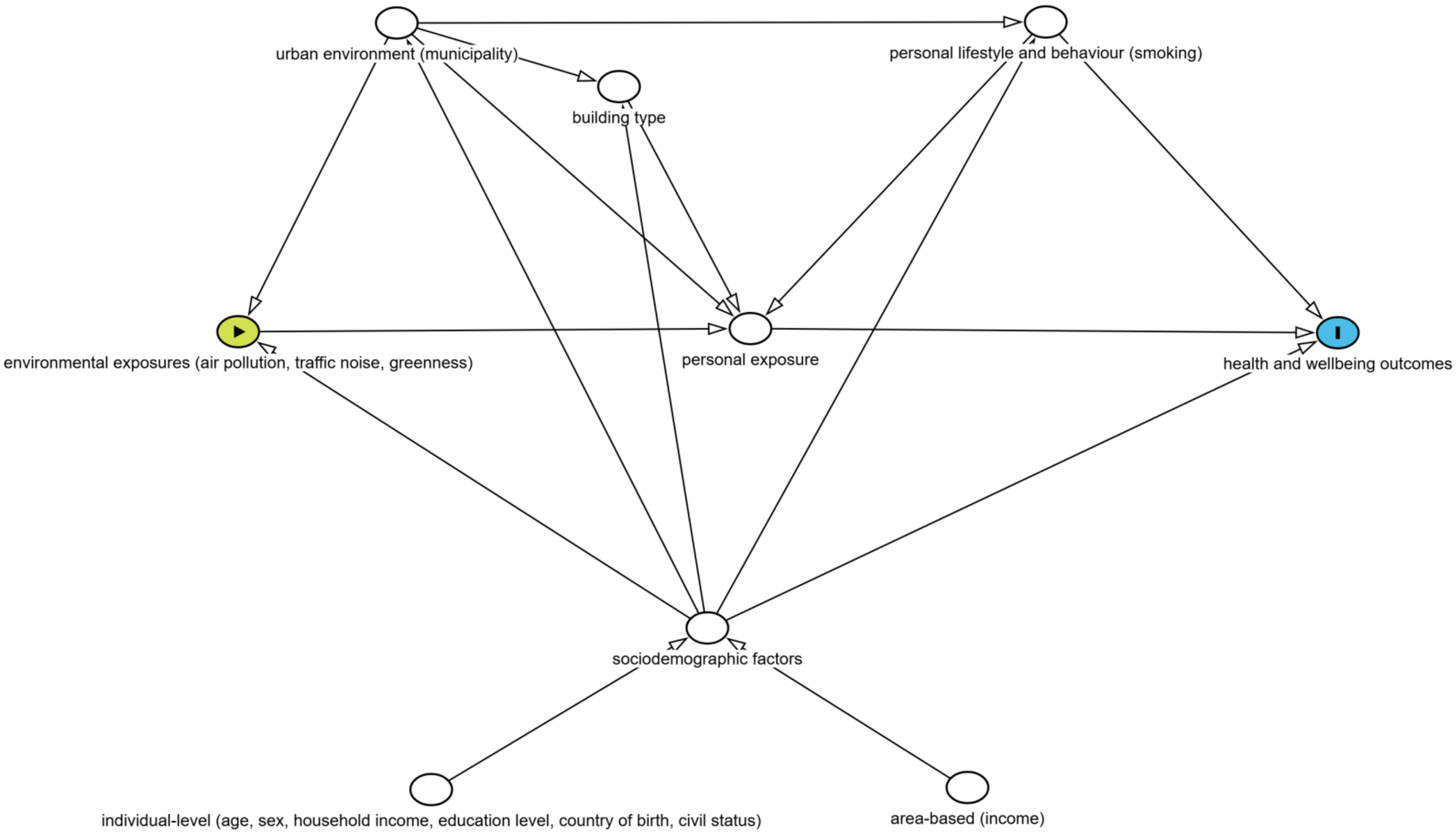

Table S0. Exposure assessment

Exposure	Exposure assessment method
**Air pollution**	Air pollution exposure was assessed as annual mean concentration of $PM_{2.5}$, PM□□, and NO□ using high-resolution dispersion modelling for the year 2022. Local emission sources (traffic, residential heating, shipping, and industry) were modelled using Gaussian dispersion models at fine spatial resolutions, as detailed in previous studies [59], while long-range transported pollution was estimated using chemical transport modelling on a coarser 15 km grid. Annual mean concentrations were assigned to participants based on their residential addresses for the year 2022. [59]
**Road traffic noise**	Road traffic noise exposure was assessed separately for road, railway, and aircraft noise. Road-traffic noise exposure was modelled using a modified version of the Nordic prediction model (Nord2000) for the year 2020. The model incorporated detailed input data including 3D terrain, road network characteristics, daily traffic volumes (including heavy vehicles), speed limits, and ground surface types. Reflection and shielding effects were estimated using a Ground Space Index based on building density. Noise levels (L_{den}) were calculated at the most exposed façade at the participants' residential address.[60]
**Railway noise**	Railway noise exposure was assessed using strategic noise maps from the Swedish Transport Administration for intercity railways and noise maps from local Stockholm authorities for light rail systems, representing conditions in 2018.[61] These data were combined with Environmental Noise Directive (END) railway noise maps for 2022, available for parts of Stockholm County.[62] When available, END 2022 data were used preferentially as the most recent exposure information. Exposure was assigned at the residential address level. For the 2018 datasets, noise contributions from multiple railway sources were harmonized into L_{den} using correction

	coefficients from Brink et al. (2018) [63] and combined using an energy summation approach.[64], This provided consistent address-level estimates of long-term railway noise exposure with high spatial resolution.
**Aircraft noise**	Aircraft noise exposure was esitmated using a novel trajectory-based framework based on high-resolution, real-world flight tracking data from the OpenSky Network.[64] These data consist of actual flight trajectories provided as aircraft positions, speeds, and altitudes over time, from which prescribed flight procedures were optimised with a mixed analysis-synthesis approach to estimate engine thrust profiles of individual flights and, ultimately, single-event noise levels.[65] The noise modelling was performed using the flight tracking data of 48 representative days in 2022, selected specifically to capture the typical annual flight patterns around Stockholm. Modelled single-event noise levels were aggregated into annual weighted average noise indicators, including L_{night}, L_{day}, and $L_{Aeq,24h}$. The modelling was conducted separately for Bromma and Arlanda airports, using for each over 17,000 receivers placed on a 60 x 60 km^2 grid and accounting for terrain elevation and noise shielding effects.[66] For addresses exposed to both airports, a combined exposure was calculated by cumulating the sound energy estimated from the two separate noise computations.
**Greenness**	Residential greenness exposure was estimated using the Normalized Difference Vegetation Index (NDVI), derived from satellite imagery for the years 2022 and 2023. NDVI was used as an indicator of surrounding vegetation density and does not directly measure greenspace accessibility, quality, or usability..[67] For each participant, NDVI values were averaged within a 300 m radial buffer around the residential address, using the annual maximum value for each year. This buffer reflects immediate residential surroundings and a typical walking distance. NDVI estimates were based on 25 × 25 m grid resolution across Sweden.[68]

Table S1. Coding of outcome variables

Manuscript outcomes	Original question from the survey (translated from Swedish)	Combination/dichotomization
Good general health (yes/no)	How to you rate your general health?<br>*Very good, good, moderate, poor, very poor.*	Yes = *very good* OR *good*
Poor outdoor air quality (yes/no)	How do you think the air quality is in the proximity of your home?<br>*Very good, good, moderate, poor, very poor.*	Yes = *poor* OR *very poor*
Symptoms from eyes, nose, and/or throat (yes/no)	In the last 3 months, how often did you have the following complaints?<br>- itching, burning, or irritation in the eyes: *at least once a week, more rarely, never*<br>- irritated, stuffy, or runny nose: *at least once a week, more rarely, never*<br>- hoarseness or dry throat: *at least once a week, more rarely, never*<br>- cough: *at least once a week, more rarely, never*	Yes = <u>at least one</u> of the questions answered with *at least once a week*
Bronchitis (yes/no)	Do you have one of the following diseases:<br>- chronic bronchitis: *yes, no*	Yes = *yes*
Asthma (yes/no)	- Do you have asthma as diagnosed by a doctor? *Yes, no*<br>- In the past 12 months, have you used any asthma medication? *Yes (daily), Yes (if needed), no*	Yes = first question answered with *yes* AND second question answered with *yes OR yes (daily)* OR *yes (if needed)*

High noise annoyance (yes/no)	In the past 12 months, in or in proximity of your home, how often have you been annoyed by noise or other sounds from:<br>- road traffic: *very much, much, moderate, little, not at all*<br>- rail traffic: *very much, much, moderate, little, not at all*<br>- air traffic: *very much, much, moderate, little, not at all*	Yes = *very much* OR *much*, respectively
Disturbance of rest and relaxation by traffic (yes/no)	How often does traffic noise (road, rail, or air traffic), in or in proximity of your home, cause the following complaints:<br>- difficult to rest and relax: *daily, every week, every week in some periods of the year, more rarely, never*	Yes = *daily* OR *every week* OR *every week in some periods of the year*
Sleep disturbance by traffic (yes/no)	How often does traffic noise (road, rail, or air traffic), in or in proximity of your home, cause the following complaints:<br>- difficult to sleep: *daily, every week, every week in some periods of the year, more rarely, never*<br>- being woken up: *daily, every week, every week in some periods of the year, more rarely, never*<br>- worsened sleep quality: *daily, every week, every week in some periods of the year, more rarely, never*	Yes = <u>at least one</u> of the 3 questions answered with *daily* OR *every week* OR *every week in some periods of the year,*
Nuisance while on terrasse or balcony by traffic (yes/no)	How often does traffic noise (road, rail, or air traffic), in or in proximity of your home, cause the following complaints:<br>- difficult to spend time on balcony/terrasse: *daily, every week, every week in some periods of the year, rarely, never*	Yes = *daily* OR *every week* OR *every week in some periods of the year*

General stress-symptoms (e.g. fatigue, headache) (yes/no)	In the last 3 months, how often did you have the following complaints?<br>- fatigue: *at least once a week, more rarely, never*<br>- headache: *at least once a week, more rarely, never*	Yes = at least one question answered with *at least once a week*
Much time spent outdoors on balcony or terrasse (in summer) (yes/no)	-Does your home have access to an outdoor space or garden? *Yes, no*<br>-How often do you spend time in the outdoor space or garden in summer? *Daily, several times a week, once a week, more rarely, never*	Yes = first question answered with *yes* AND second question answered with *daily OR several times a week OR once a week*
Pollen allergy (yes/no)	Are you hypersensitive or allergic towards the following and how serious are your complaints:<br>- Pollen (leaf trees, grass, mugwort or other plants): *Yes (strong complaints), yes (light complaints), yes (but less to no complaints when medicated), no*	Yes = *Yes (strong complaints)* OR *yes (light complaints)* OR *yes (but less to no complaints when medicated)*
Frequent greenspace visits (yes/no)	- How often do you visit parks, greenspace or other nature in summer? *Daily, once/several times a week, once/several times a month, once/several times a year, never*<br>- How often do you visit parks, greenspace or other nature in winter? *Daily, once/several times a week, once/several times a month, once/several times a year, never*	Yes = at least one of the questions answered with *daily* OR *once/several times a week*
High indoor temperatures (in summer)	How often do you complain that your home is one of the following:	Yes = *Daily* OR *at least once a week week*

- too warm in summer? *Daily, at least once a week, more rarely, never*

Figure S1. Distribution of air pollution, road traffic noise, and greenness (red line = median).

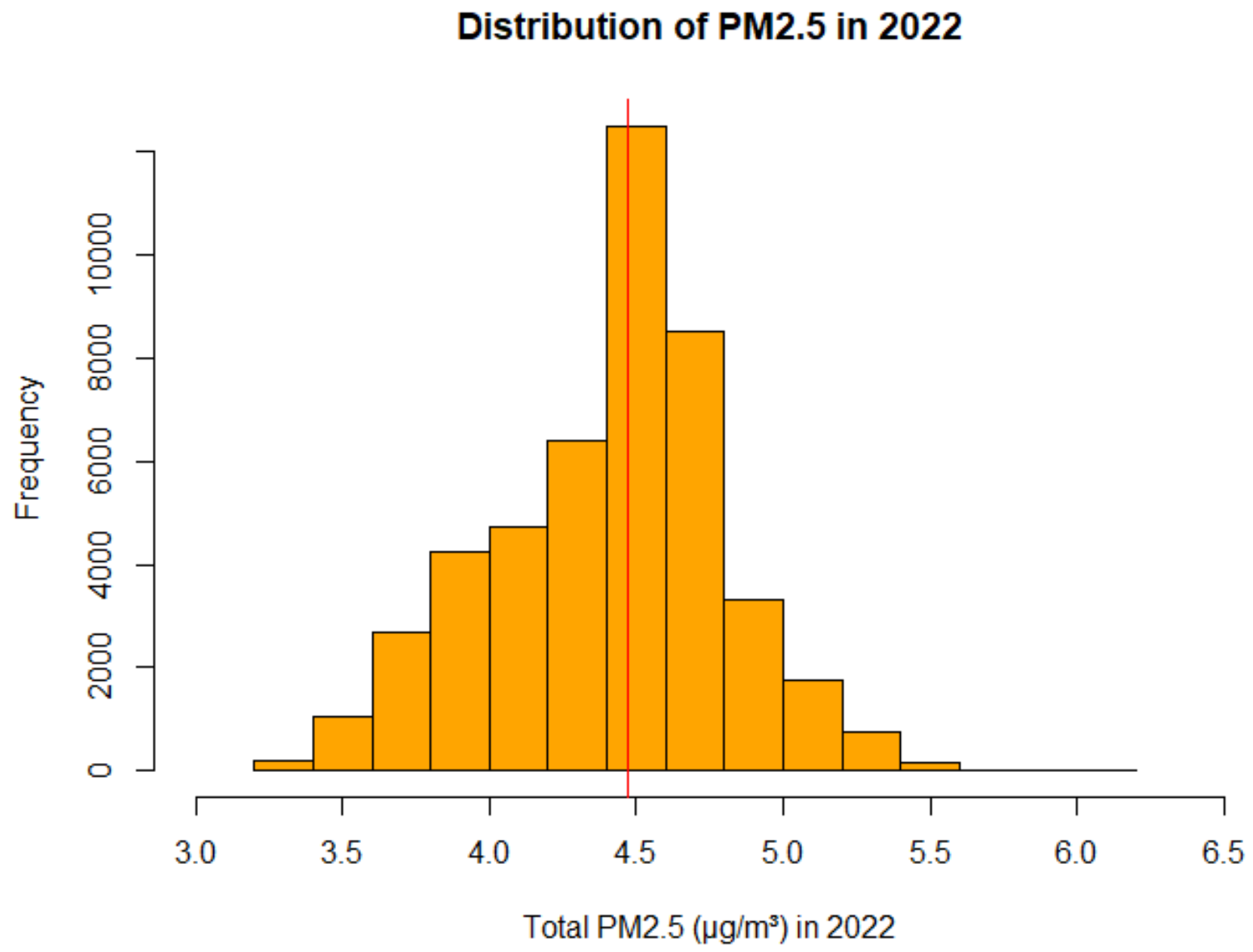

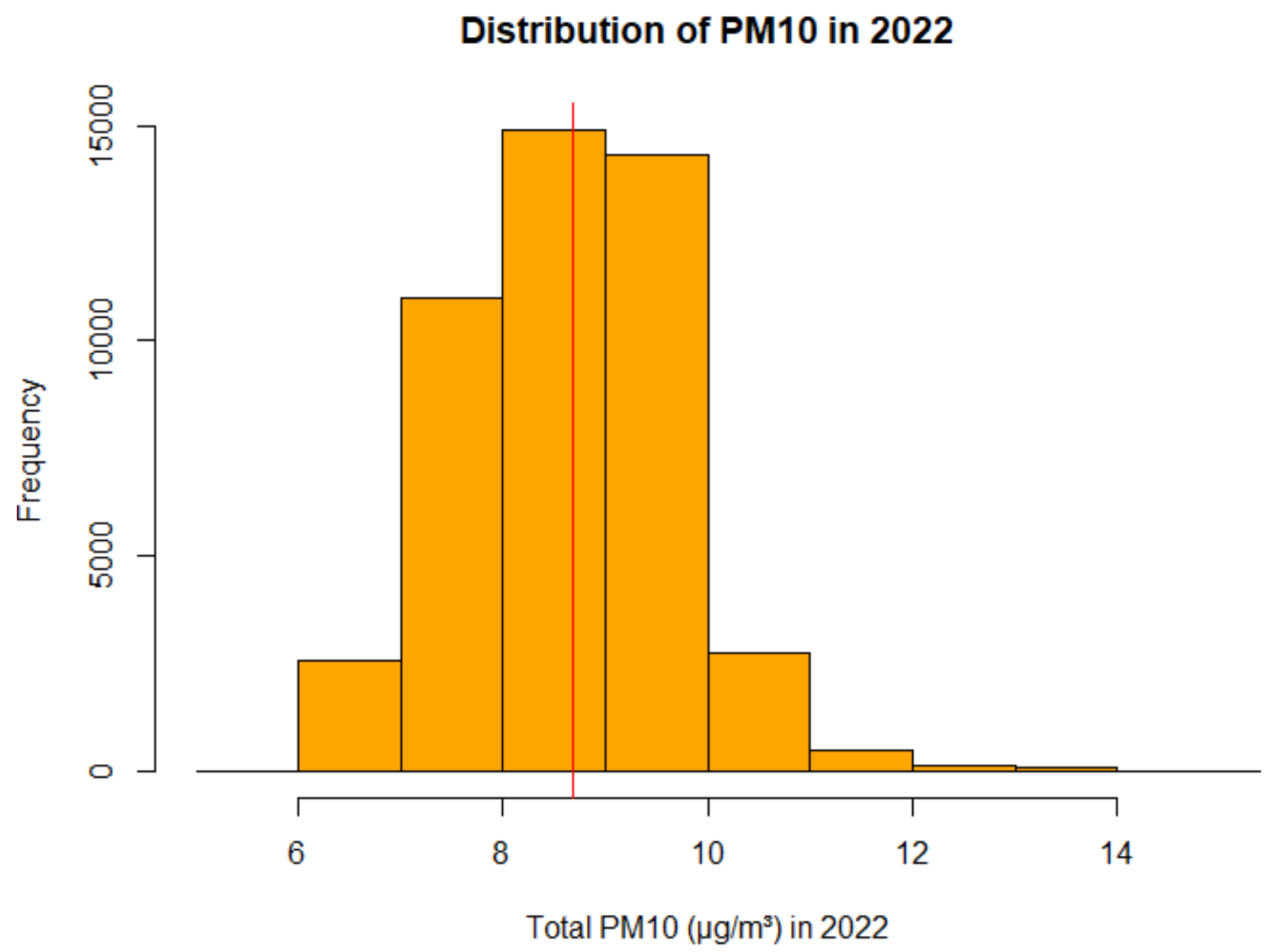

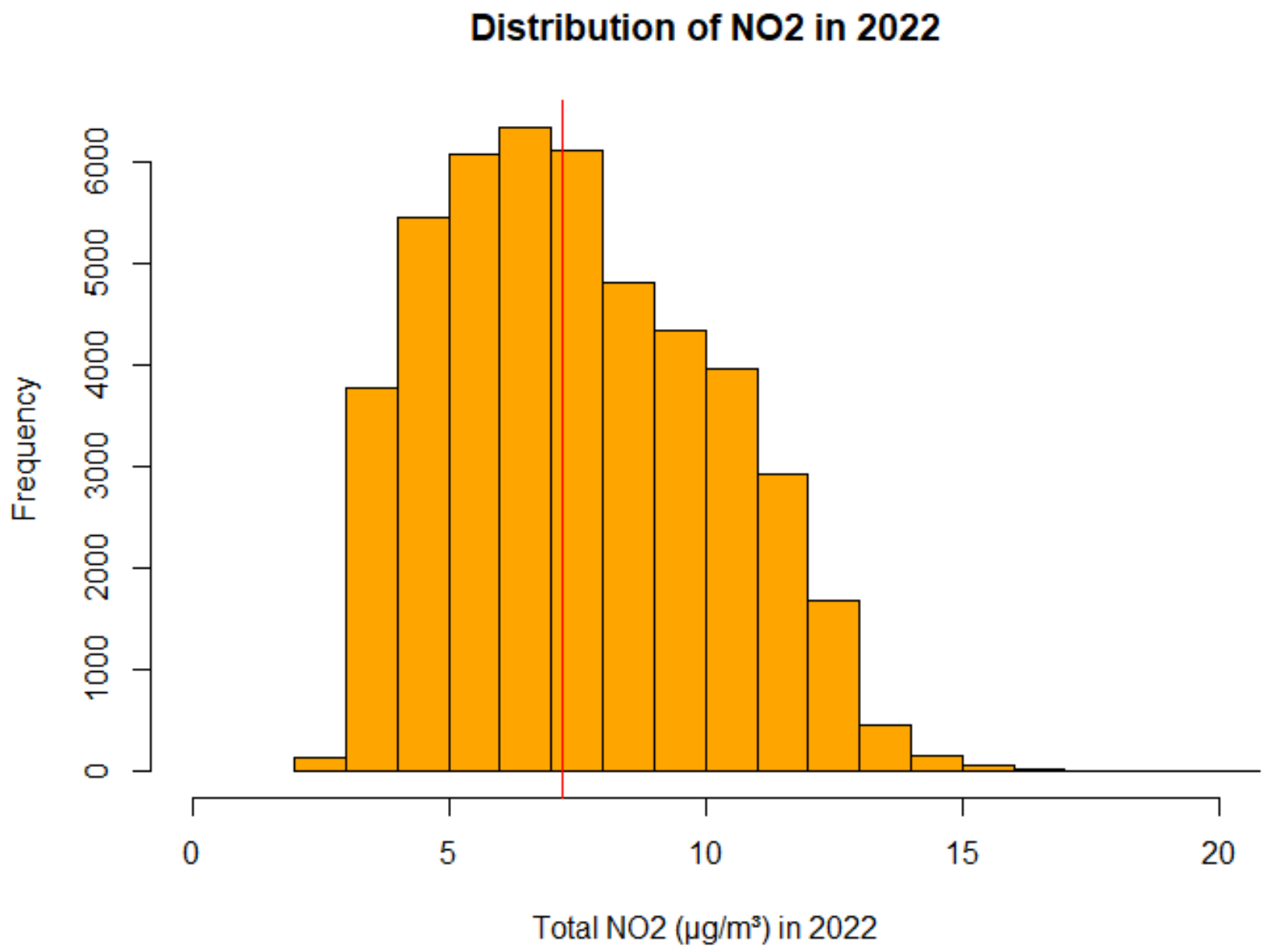

Distribution of NO2 in 2022
Frequency
0
1000
2000
3000
4000
5000
6000
0
5
10
15
20
Total NO2 (µg/m³) in 2022

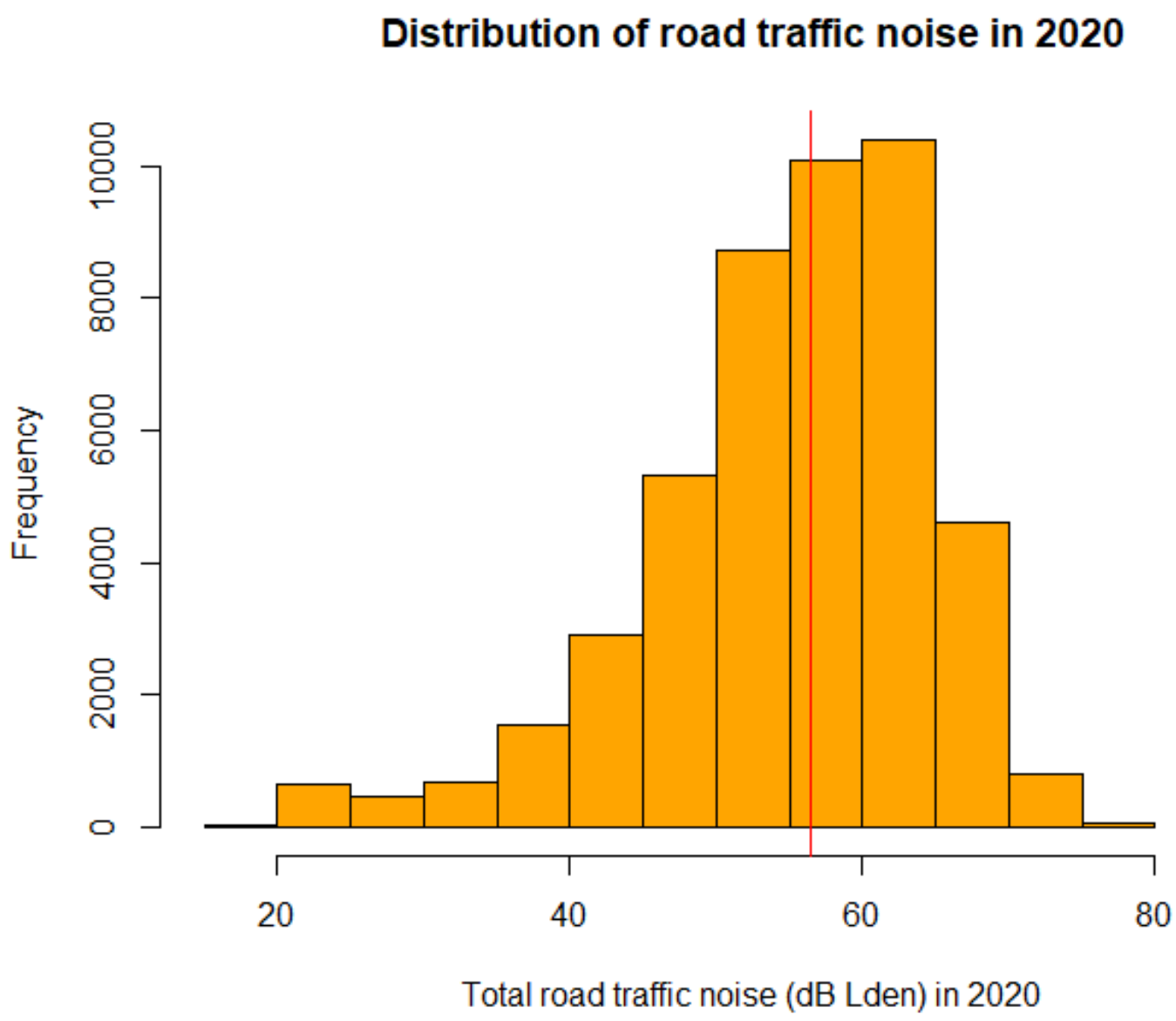

Distribution of road traffic noise in 2020
Frequency
0
2000
4000
6000
8000
10000
20
40
60
80
Total road traffic noise (dB Lden) in 2020

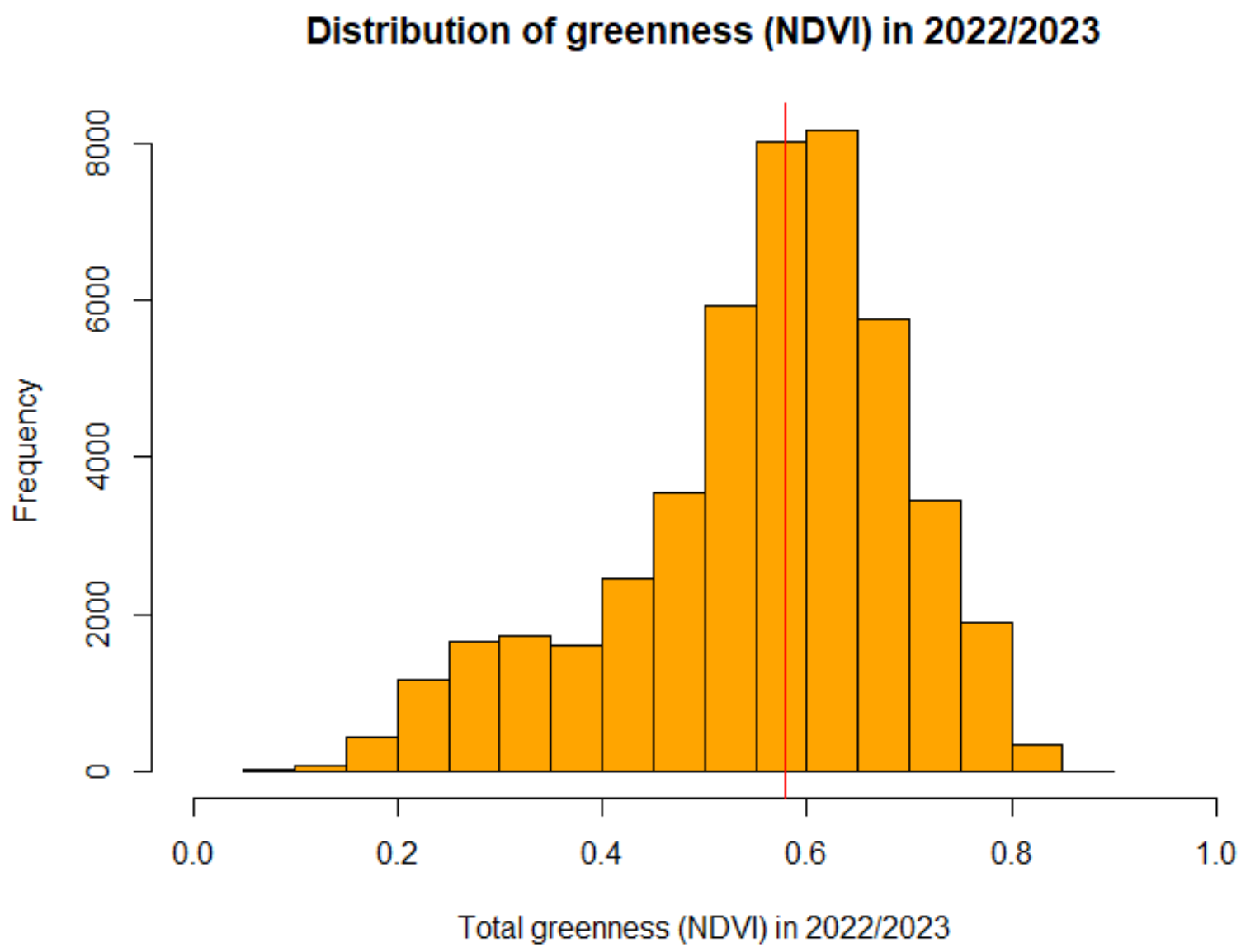
Distribution of greenness (NDVI) in 2022/2023
Frequency
0
2000
4000
6000
8000
0.0
0.2
0.4
0.6
0.8
1.0
Total greenness (NDVI) in 2022/2023

Figure S2. Correlation matrix of exposure variables (Pearson correlation).

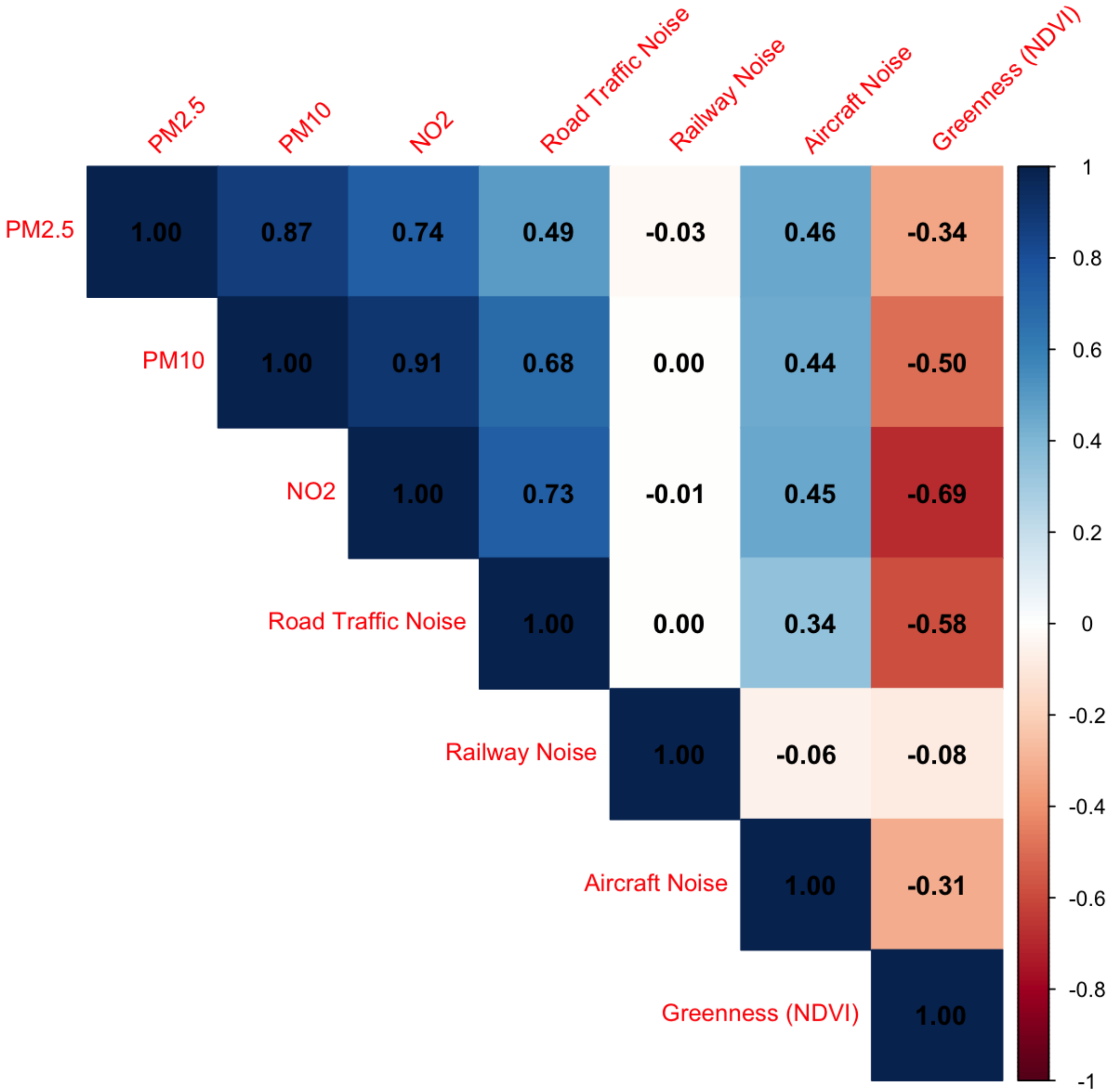

Table S2. Proportion of people exposed above respective cut-off values, stratified by income, education, country of birth and building type - in the *weighted* study sample.

Exposure	Overall	Income[a]		Education[a]		Country of birth[a]		Building type[a]	
		High[e]	Low[e]	High[f]	Low[f]	Domestic	Foreign	House	Apartment building
		N = 23,137	N = 23,137	N = 22,175	N = 23,919	N = 37,975	N = 7,951	N = 20,662	N = 24,043
**Air pollution[b]**									
$PM_{2.5}$	58.2%	61.1%	55.5%	65.7%	52.7%	59.1%	55.7%	49.8%	63.4%
PM_{10}	59.5%	58.7%	60.3%	67.7%	53.5%	59.0%	60.6%	37.1%	72.2%
NO_2	59.9%	57.7%	61.8%	68.5%	53.6%	59.3%	61.3%	31.0%	76.1%
**Traffic noise[c]**									
Road	72.3%	67.1%	77.0%	75.9%	69.5%	70.5%	76.4%	44.8%	87.7%
Railway	8.54%	8.15%	8.88%	9.60%	7.73%	8.42%	8.81%	4.00%	10.9%
Aircraft	3.94%	4.01%	3.87%	3.95%	3.93%	3.94%	3.89%	4.37%	3.77%
**Greenness[d]**									
NDVI	77.50%	78.1%	76.9%	72.0%	81.4%	76.5%	80.2%	98.7%	66.5%

[a] %, Pearson's Chi-squared test, less than 9% missing for each variable

[b] Air pollutants as binary variables. Cut- offs (high) in µg/m³ at respective medians – $PM_{2.5} \geq 4.47$, $PM_{10} \geq 8.68$, $NO_2 \geq 7.23$.

[c] Noise as binary variables. Cut-offs (high) in dB L_{den}, according to WHO limits for traffic noise – road ≥ 53, railway ≥ 54, aircraft ≥ 45.

[d] Greenness as binary variable. Cut-off (high) in Normalised Difference Vegetation Index (NDVI) at ≥ 0.45.

[e] low income = below or equal median (≤652,552); high income = above median (>652,552)

[f] low education = no university degree; high education = university degree

Table S3. Distribution of health and wellbeing outcomes of the study sample.

**Characteristics**	**N** [a]	**Overall,** N = 46,282 [b]
**Good general health**	45,957	
Yes		34,006 (74%)
**Poor outdoor air quality**	45,837	
Yes		2,682 (5.9%)
**Symptoms from eyes, nose, and/or throat**	46,053	
Yes		14,443 (31%)
**Bronchitis**	44,821	
Yes		849 (1.9%)
**Asthma**	45,824	
Yes		3,966 (8.7%)
**High annoyance due to road traffic noise**	45,872	
Yes		2,710 (5.9%)
**High annoyance due to railway traffic noise**	45,803	
Yes		590 (1.3%)
**High annoyance due to aircraft traffic noise**	45,801	
Yes		1,464 (3.2%)
**Disturbance of rest and relaxation**	45,778	
Yes		3,263 (7.1%)
**Sleep disturbance by traffic**	45,783	
Yes		3,752 (8.2%)
**Nuisance while on terrasse or balcony**	45,802	
Yes		2,636 (5.8%)
**General stress-symptoms (e.g. fatigue, headache)**	46,060	
Yes		21,433 (47%)
**Much time spent outdoors on balcony or terrasse (in summer)**	39,066	
Yes		33,704 (86%)
**Pollen allergy**	46,023	
Yes		18,603 (40%)
**Frequent greenspace visits**	46,000	
Yes		37,166 (81%)
**High indoor temperatures (in summer)**	45,707	
Yes		14,879 (33%)

[a] less than 9% missing for each variable
[b] n (%); Median (IQR)

Table S4. Associations between environmental exposures and indicators of health and wellbeing (all), without area-based (DeSo) income as covariate.

	**Air pollution**[a, b] (µg/m³)			**Traffic noise**[a, c] (dB L_{den})			**Greenness**[a, d] (NDVI)
**Outcomes**	**$PM_{2.5}$**	**PM_{10}**	**NO_2**	**Road**	**Railway**	**Aircraft**	**NDVI**
Good general health	1.06 (0.99-1.13)	1.05 (0.98-1.13)	0.99 (0.92-1.06)	0.91 (0.86-0.95)	0.98 (0.90-1.07)	1.11 (0.98-1.26)	0.95 (0.89-1.01)
Poor outdoor air quality	1.42 (1.26-1.60)	2.70 (2.32-3.15)	3.22 (2.75-3.79)	-	-	-	-
Symptoms from eyes, nose, and/or throat	0.93 (0.88-0.99)	1.00 (0.93-1.06)	1.09 (1.02-1.16)	-	-	-	0.95 (0.90-1.00)
Bronchitis	1.02 (0.84-1.24)	1.06 (0.86-1.32)	1.15 (0.93-1.43)	-	-	-	-
Asthma	1.02 (0.93-1.13)	0.99 (0.89, 1.10)	1.01 (0.91-1.13)	-	-	-	-
High annoyance	-	-	-	4.64 (4.07-5.32)	8.19 (6.79-9.88)	7.29 (6.27-8.45)	-
Disturbance of rest and relaxation	-	-	-	1.81 (1.64-2.00)	1.37 (1.22-1.55)	1.81 (1.54-2.11)	-
Sleep disturbance by traffic	-	-	-	1.81 (1.65-2.00)	1.47 (1.31-1.64)	1.43 (1.22-1.68)	-
Nuisance while on terrasse or balcony	-	-	-	2.42 (2.15-2.73)	1.75 (1.54-1.97)	2.26 (1.92-2.65)	-
General stress-symptoms (e.g. fatigue, headache)	-	-	-	1.04 (0.99-1.09)	1.05 (0.98-1.13)	0.95 (0.85-1.06)	0.99 (0.94-1.05)
Much time spent outdoors on balcony or terrasse (in summer)	-	-	-	0.33 (0.30-0.36)	0.75 (0.68-0.83)	1.24 (1.06-1.45)	2.78 (2.59-2.99)
Pollen allergy	-	-	-	-	-	-	1.02 (0.97-1.07)
Frequent greenspace visits	-	-	-	-	-	-	1.09 (1.02-1.16)
Perceived high indoor temperatures (in summer)	-	-	-	1.13 (1.08-1.19)	1.18 (1.09-1.27)	0.98 (0.87-1.10)	0.83 (0.79-0.88)

[a] Odds ratios with confidence intervals (95%).
Logistic regression models adjusted for sex, age, country of birth, household income, education level, civil status, smoking, and municipality. Less than 9% missing for each variable

[b] Air pollutants as binary variables. Cut- offs (high) in µg/m³ at respective medians – $PM_{2.5}$ ≥ 4.47, PM_{10} ≥ 8.68, NO_2 ≥ 7.23.

[c] Noise as binary variables. Cut-offs (high) in dB L_{den}, according to WHO limits for traffic noise – road ≥ 53, railway ≥ 54, aircraft ≥ 45.

[d] Greenness as binary variable. Cut-off (high) in Normalised Difference Vegetation Index (NDVI) at ≥ 0.45.

References

59. Kilbo Edlund K, Andersson EM, Asker C, et al. Long-term ambient air pollution and coronary atherosclerosis: Results from the Swedish SCAPIS study. Atherosclerosis. 2024;397. doi:10.1016/j.atherosclerosis.2024.117576

60. Murzabekov M, Persson Å, Asker C, et al. Road-traffic noise exposure and coronary atherosclerosis in the Swedish CArdioPulmonary bioImage Study (SCAPIS). Environmental Epidemiology. 2024;8(5):e344. doi:10.1097/EE9.0000000000000344

61. Stockholms stad. Bullerkartan 2022. 2024. Accessed January 22, 2026. https://open-data-sthlm-miljo.hub.arcgis.com/maps/ce2b61853e594b7fa70de53eed7e9ef2/about

62. European Commission. Environmental Noise Directive. 2022. Accessed January 22, 2026. https://environment.ec.europa.eu/topics/noise/environmental-noise-directive_en#

63. Brink M, Schäffer B, Pieren R, Wunderli JM. Conversion between noise exposure indicators Leq24h, LDay, LEvening, LNight, Ldn and Lden: Principles and practical guidance. Int J Hyg Environ Health. 2018;221(1):54-63. doi:10.1016/j.ijheh.2017.10.003

64. Pretto M, Dorbolò L, Giannattasio P, Zanon A. Aircraft operation reconstruction and airport noise prediction from high-resolution flight tracking data. Transp Res D Transp Environ. 2024;135. doi:10.1016/j.trd.2024.104397

65. Pretto M, Giannattasio P, De Gennaro M. Mixed analysis-synthesis approach for estimating airport noise from civil air traffic. Transp Res D Transp Environ. 2022;106. doi:10.1016/j.trd.2022.103248

66. Pretto M, Giannattasio P, De Gennaro M, Zanon A, Kuehnelt H. Forecasts of future scenarios for airport noise based on collection and processing of web data. European Transport Research Review. 2020;12(1). doi:10.1186/s12544-019-0389-x

67. Sellers PJ. Canopy reflectance, photosynthesis and transpiration. Int J Remote Sens. 1985;6(8):1335-1372. doi:10.1080/01431168508948283

68. Lõhmus M, Pyko A, Georgelis A. Tillgång till Bostadsnära Grönska i Befolkningen Exponering, Utsatta Grupper Och Besvär. 2022. www.camm.sll.se
